# Multipath Curved Planar Reformations of Peripheral CT Angiography: Diagnostic Accuracy and Time Efficiency

**DOI:** 10.1007/s00270-017-1846-3

**Published:** 2017-12-07

**Authors:** Markus M. Schreiner, Hannes Platzgummer, Sylvia Unterhumer, Michael Weber, Gabriel Mistelbauer, Eduard Groeller, Christian Loewe, Ruediger E. Schernthaner

**Affiliations:** 1 Section of Cardiovascular and Interventional Radiology, Department of Biomedical Imaging and Image-Guided Therapy, Medical University of Vienna, Waehringer Guertel 18-20, 1090 Vienna, Austria; 2 Institute of Computer Graphics and Algorithms, Technical University of Vienna, Favoritenstraße 9-11, 1040 Vienna, Austria

**Keywords:** PAD, CTA, 3D reformation, mpCPRs

## Abstract

**Objectives:**

To compare diagnostic performance and time efficiency between 3D multipath curved planar reformations (mpCPRs) and axial images of CT angiography for the pre-interventional assessment of peripheral arterial disease (PAD), with digital subtraction angiography as the standard of reference.

**Methods:**

Forty patients (10 females, mean age 72 years), referred to CTA prior to endovascular treatment of PAD, were prospectively included and underwent peripheral CT angiography. A semiautomated toolbox was used to render mpCPRs. Twenty-one arterial segments were defined in each leg; for each segment, the presence of stenosis > 70% was assessed on mpCPRs and axial images by two readers, independently, with digital subtraction angiography as gold standard.

**Results:**

Both readers reached lower sensitivity (Reader 1: 91 vs. 94%, *p* = 0.08; Reader 2: 89 vs. 93%, *p* = 0.03) but significantly higher specificity (Reader 1: 94 vs. 89%, *p* < 0.01; Reader 2: 96 vs. 95%, *p* = 0.01) with mpCPRs than with axial images. Reader 1 achieved significantly higher accuracy with mpCPRs (93 vs. 91%, *p* = 0.02), and Reader 2 had similar overall accuracy in both evaluations (94 vs. 94%, *p* = 0.96). Both readers read mpCPRs significantly faster than axial images (Reader 1: 5′45″ based on mpCPRs vs. 7′40″ based on axial images; Reader 2: 4′41″ based on mpCPRs vs. 6′57″ based on axial images; *p* < 0.01).

**Conclusions:**

mpCPRs are a promising 3D reformation technique that facilitates a fast assessment of PAD with high diagnostic accuracy.

**Electronic supplementary material:**

The online version of this article (10.1007/s00270-017-1846-3) contains supplementary material, which is available to authorized users.

## Introduction

Peripheral arterial disease (PAD) and its associated morbidity place a significant burden on both patients and healthcare systems. Based on demographic projections and PAD particularly affecting the elderly [1, 2], the incidence of PAD is expected to further increase [3]. While the diagnosis is made clinically, the guidelines of the Cardiovascular and Interventional Radiological Society of Europe on endovascular treatment in aortoiliac arterial disease recommend a comprehensive radiological assessment for accurate treatment planning [4]. Digital subtraction angiography (DSA) is still considered the standard of reference because it offers the highest spatial and temporal resolution. However, over the last 20 years, computed tomography angiography (CTA) has evolved into an accurate and cost-effective imaging alternative [5–7], with high clinical impact for patient management [8, 9]. However, the improved spatial resolution has resulted in an increasing number of axial slices [10]. Thus, axial image evaluation is a progressively time-consuming and cumbersome task in the clinical routine [11], which bears the risk of missing small pathologies [12, 13]. 3D reformations provide a better overview and facilitate the visualization of complex anatomical structures. This might result in superior diagnostic performance and shorter evaluation times. For treatment planning, not only the severity, but also length and number of stenoses within a vascular segment are essential parameters according to the Trans-Atlantic Inter-Society Consensus (TASC) guidelines [14]. By providing an angiogram-like view of the peripheral vessels, 3D reformations facilitate the assessment of all treatment-relevant parameters. Thus, many different techniques have been developed. Maximum intensity projections (MIPs) provide a distortion-free display of the arterial tree, but are inherently unsuitable for the assessment of calcified vessels [15]. In addition, automated algorithms for bone segmentation, which is a prerequisite for vascular MIPs, tend to fail when vessels are not separated by soft tissue from the bones, which happens frequently in the path of the anterior tibial artery [16, 17]. Portugaller et al. [18] showed that semitransparent volume rendering (STVR) of CTA provided accuracy superior to that of MIPs in calcified vessels. However, both MIP and STVR failed to provide sufficient diagnostic performance when used alone [19], so that these techniques could be used only as a supplement to axial images. Curved planar reformations (CPRs) were shown to depict the cross-sectional profile of a vessel along its length while preserving the relative X-ray attenuation information [20], resulting in a higher accuracy than MIPs in calcified vessel segments [18]. However, CPRs are limited to a single vessel path, by design [21]. Multipath CPRs (mpCPRs) were developed to overcome this limitation [22]. These reformations were shown to facilitate the assessment of the peripheral arterial tree according to the TASC guidelines [14], when used in conjunction with axial images [23]. However, the diagnostic accuracy of CTA for the assessment of PAD, based exclusively on mpCPRs, has not been evaluated. Thus, the purpose of this study was to compare the diagnostic performance and the time efficiency between mpCPRs and axial images of peripheral CTA for the assessment of PAD, with DSA as the standard of reference.

## Materials and Methods

This prospective single-center study was approved by the local ethics committee. Written informed consent was obtained from all patients prior to recruitment. Patients were referred from the Division of Angiology and Vascular Surgery. The following served as inclusion criteria: Rutherford Category 2–6; age ≥ 18 years; and normal TSH levels. The following served as exclusion criteria: patient on dialysis (estimated glomerular filtration rate < 30 ml/min); pregnancy; and breastfeeding. The time interval between CTA and DSA had to be equal to or less than 30 days to avoid a bias attributable to disease progression. Patients who did not ultimately undergo DSA, or who exceeded the required 30-day interval between CTA and DSA, were excluded from the study.

### Multidetector CT Angiography

All patients underwent CTA on a second-generation, dual-source, multidetector CT scanner (Somatom Definition Flash, Siemens Medical Systems, Erlangen, Germany). A programmable power injector with a dedicated injection protocol (OptiBolus, Covidien, Austria) was used to administer 90 ml of a low osmolar, non-ionic iodinated contrast agent (ioversol, Optiray 350, Covidien, Austria). In this protocol, a monophasic contrast agent injection with an exponentially decreasing flow rate (3.5–2.6 ml/s) over 35 s was followed by a 35-ml saline flush at a flow rate of 2.6 ml/s [24]. Starting 10 s after the contrast injection, the reference scan was repeated each second in the aorta at the origin of the renal arteries until the enhancement reached 150 Hounsfield units (HU). The final scan was initiated after another 4-s delay, covering a volume from the renal arteries to the mid-foot. Whereas the tube voltage was set to 80 kV in all patients, the reference for tube current modulation was set to 120 and 150 mAs for patients with a BMI smaller than or equal to 25 or a BMI larger than 25, respectively [25]. Iterative image reconstruction (SAFIRE strength level 3, Siemens Medical Systems, Erlangen, Germany) was applied during the reconstruction of 1.5-mm-thick axial slices.

### Image Post-processing

The axial slices were transferred over a Digital Imaging and Communications in Medicine (DICOM) network to our prototype 3D workstation [22]. Image post-processing was routinely performed by the CT technologist in charge on the day of each CT scan. For the creation of MIPs, a semiautomated bone segmentation algorithm was applied to suppress bone structures. For the creation of mpCPRs, the extraction of the arterial centerline trees between a starting point in the infra-renal aorta and six end points located in the most distal portions of the bilateral dorsal and plantar artery of the foot, as well as the peroneal arteries, was based on a semiautomated vessel tracking and centering algorithm. In patients without occlusions, the technologists had to place only a few control points and the software automatically identified the vessel segments in between these control points. However, through the course of an occlusion, multiple control points were required as the vessel growth algorithm is based on vascular enhancement. Thus, the user interaction time ranged from 2 to 25 min, based on the number and length of occlusions. For both reformation techniques, 21 images were generated fully automated over a viewing range of 180° (from right lateral [− 90°] through anteroposterior [0°] to left lateral [+ 90°] viewing angles) in 9° intervals and saved in DICOM format to preserve the original CT attenuation information (supplemental material). Of note, the images were rendered using the full resolution along the *z*-axis. In other words, if a dataset consisted of 2000 axial images, the reformations had a resolution of 512 × 2000 voxels.

### Digital Subtraction Angiography

All DSA studies were performed routinely on an Axiom Artis, Angiostar or an ArtisZeego Digital Angiography System (Siemens Systems, Erlangen, Germany) via antegrade or retrograde puncture of a common femoral artery. A low osmolar, non-ionic, iodinated contrast agent (Ioversol, Optiray 350, Covidien, Austria) at a concentration of 350 mg iodine per ml was used.

### Image Analysis

**Fig. 1:**
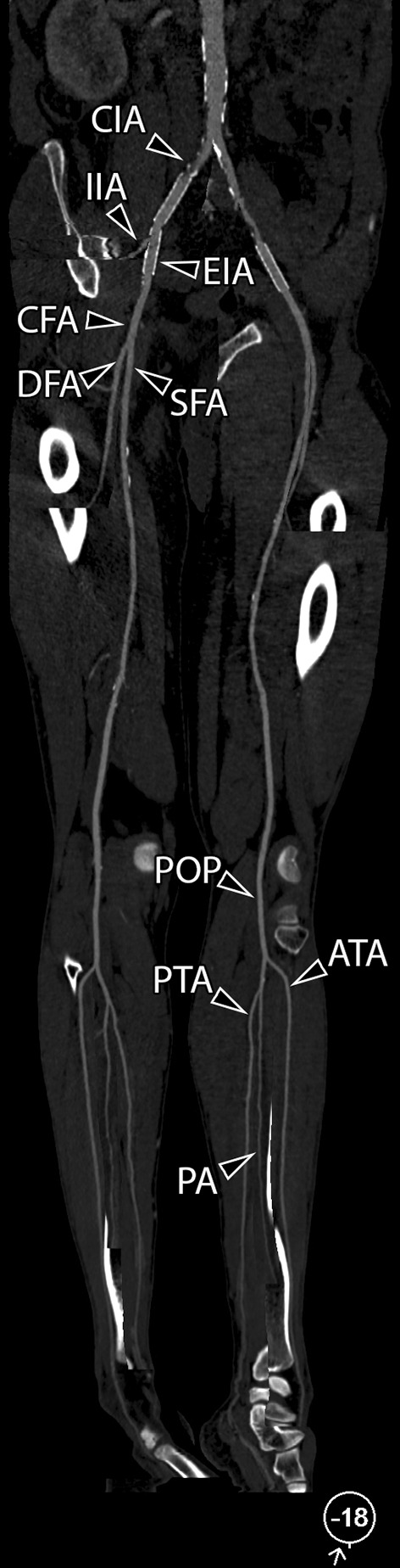
A 52-year-old male patient with a history of stent implantation in the right common iliac artery and both external iliac arteries was referred to interventional radiology with PAD category 2 according to the Rutherford classification. The arterial tree of each leg was divided into 21 segments, as demonstrated on mpCPR at a viewing angle of − 18° (right-oblique view): *CIA* common iliac artery, *EIA* external iliac artery, *IIA* internal iliac artery, *CFA* common femoral artery, *DFA* deep femoral artery, *SFA* superficial femoral artery, *POP* popliteal artery, *ATA* anterior tibial artery, *TPT* tibioperoneal trunk, *PA* peroneal artery, *PTA* posterior tibial artery. SFA, POP, ATA, PA, and PTA were subdivided into three segments: proximal, middle, and distal

Image analysis was performed on a picture archiving and communication system (PACS) workstation (IMPAX EE R20, Agfa Healthcare N.V., Mortsel, Belgium). The arterial tree of each leg was divided into 21 segments (Fig. 1). Furthermore, each segment was assigned to one of three different groups (iliac arteries, femoro-popliteal arteries, infra-popliteal arteries) according to their anatomical location. Two readers, one specialist-in-training (Reader 1 H.P., 5 years of experience) and one expert vascular radiologist (Reader 2, R.E.S., 10 years of experience), independently assessed CT angiography, once based on axial images and once based on mpCPRs. The study coordinator recorded the time needed for the evaluation of axial images and mpCPRs for every patient. Both readers were blinded to all patient details. DSA images, which served as the reference standard, were assessed exclusively by Reader 2. To eliminate recall bias, the evaluation of axial images and mpCR images, as well as DSA images, was separated by at least an 8-week interval each. For each segment, the most severe stenosis was assessed for hemodynamic significance (> 70%). Five additional categories were defined for segments that were not assessable due to one of the following reasons: not depicted; insufficient contrast; severe calcifications; prosthesis-related artifacts; or stent-related artifacts.

### Statistical Analysis

All statistical evaluations were performed using IBM SPSS for Windows, version 22.0.0.2 (IBM, NY, USA). Nominal data are described using absolute frequencies and percentages. The sample size calculation was performed for the accuracy assessment based on data of previous studies at our institution [8, 9, 23]. Generalized estimating equations were used to compare sensitivity, specificity, and accuracy, as well as positive predictive value and negative predictive value, of axial images and mpCPRs, with DSA as standard of reference to take into account multiple measurements per patient. Additional generalized estimating equations were used to calculate the influence of PAD stage on the diagnostic accuracy, sensitivity and specificity of axial images and mpCPRs, respectively. Absolute agreement was used as a measure for inter-reader agreement and was compared between axial images and mpCPRs using generalized estimating equations. A *p* value less than 0.05 was considered significant.

## Results

**Fig. 2:**
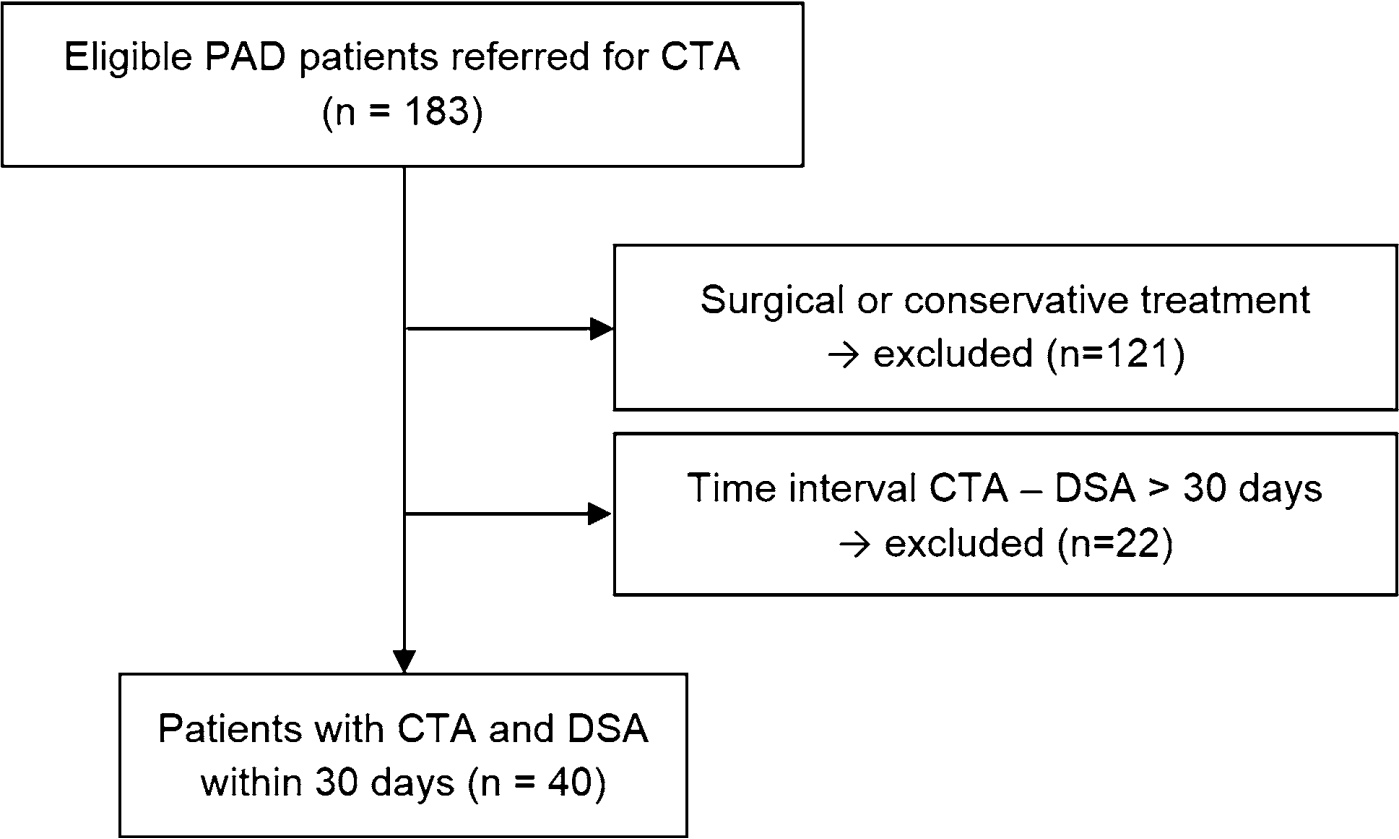
Flowchart demonstrating patient recruitment

During the study period of 12 months, 183 PAD patients were referred to CTA of the peripheral arteries. A total of 121 patients had to be excluded because no endovascular, but surgical or conservative treatment was performed after CTA. In another 22 patients, endovascular therapy was not performed within 30 days after CTA, so that the final study cohort consisted of forty patients (30 males, 10 females; mean age 72 ± 11 years; range 44–101) as displayed in Fig. 2. For these patients, the average interval between CTA and DSA was 6 days, with a range of 1–28 days. Six patients of our study cohort had a total of eight orthopedic implants, and 17 patients had a total number of 47 stents in the segments that were evaluated in this study. Demographic characteristics, including risk factors for PAD, are displayed in Table 1.

**Table 1:** Patient characteristics

No. of patients	40 (100)
Sex	
Male	30 (75)
Female	10 (25)
Age (years)^a^	72 ± 11 (44–101)
Rutherford category	
2	12 (30)
3	8 (20)
4	3 (7.5)
5	6 (15)
6	11 (27.5)
Risk factors	
Smoker	17 (43)
Hyperlipidemia	20 (50)
Hypertension	21 (53)
Diabetes	16 (40)
Coronary artery disease	10 (25)

Except where indicated, data represent numbers of patients, and numbers in parentheses are percentages

^a^Data are given as mean ± SD and range in parentheses

### Diagnostic Performance

Due to ethical considerations, the DSA procedure was not specifically adapted for study reasons. As a result, DSA did not depict the entire vasculature of both legs in each patient, but only the treatment-relevant areas, including in- and outflow. In particular, interventions were limited to one leg in 31 patients. In 16 of those patients, DSA was performed after antegrade arterial puncture of the common femoral artery, thus depicting only segments downriver of the puncture site. In the other 15 patients, retrograde puncture of the contralateral common femoral artery was followed by a crossover maneuver, which provided images of the bilateral iliac arteries as well. In the remaining nine patients, the vasculature of both legs was completely depicted by DSA. Altogether, 888 segments were depicted by DSA and could be included in the analysis.

Of these 888 segments, Reader 1 rated 44 (5.0%) and 76 (8.6%) segments non-assessable on mpCPRs and axial images, respectively (16 and 25 for low contrast, 10 and 19 due to prosthesis-related artifacts, one and seven due to stent-related artifacts, and 17 and 25 due to severe circumferential calcifications, respectively). Reader 2 rated only 25 (2.8%) and 29 (3.3%) segments non-assessable on mpCPRs and axial images, respectively (one each for low contrast, nine each due to prosthesis-related artifacts, and 15 and 19 due to severe circumferential calcifications, respectively).

Overall, both readers yielded lower sensitivity values with mpCPRs compared to axial images. However, the difference was significant only for Reader 2 (89 vs. 93%, *p* = 0.03), but not for Reader 1 (91 vs. 94%, *p* = 0.08). With regard to specificity, both readers performed significantly better using mpCPRs (Reader 1: 94 vs. 89%, *p* < 0.01; Reader 2: 96 vs. 95%, *p* = 0.01). Reader 1 also achieved significantly higher accuracy with mpCPRs (93 vs. 91%, *p* = 0.02), whereas Reader 2 had an overall accuracy that was similar with both mpCPRs and axial images (94 vs. 94%, *p* = 0.96). In addition, both readers had a higher overall positive predictive value (PPV) in the evaluation based on mpCPRs (*p* < 0.01 and *p* = 0.07). An opposite trend was observed regarding the overall negative predictive value (NPV), as both readers had lower NPVs based on mpCPRs (Reader 1: 96 vs. 98%, *p* = 0.17; Reader 2: 96 vs. 97%, *p* < 0.01). Detailed statistical parameters for each vascular territory are provided in Table 2.

**Table 2:** Diagnostic performance of CT angiography with digital subtraction angiography as the reference standard according to vascular region

	Reader 1 (H.P.)			Reader 2 (R.E.S.)		
	mpCPR	Axial	*p* value	mpCPR	Axial	*p* value
Iliac arteries						
Sensitivity	82 (9/11)	90 (9/10)	0.39	83 (10/12)	100 (12/12)	< 0.001^a^
Specificity	95 (75/79)	88 (67/76)	0.04^b^	99 (79/80)	95 (77/81)	0.20
Accuracy	93 (84/90)	88 (76/86)	0.10	97 (89/92)	96 (89/93)	0.65
PPV	69 (9/13)	50 (9/18)	0.08	91 (10/11)	75 (12/16)	0.04^b^
NPV	97 (75/77)	99 (67/68)	0.41	98 (79/81)	100 (77/77)	–^c^
Femoro-popliteal arteries						
Sensitivity	85 (74/87)	86 (74/86)	0.80	82 (75/91)	88 (82/93)	0.03^a^
Specificity	95 (305/320)	88 (271/308)	< 0.001^b^	88 (300/321)	92 (295/321)	0.13
Accuracy	93 (379/407)	88 (345/394)	< 0.001^b^	91 (375/412)	91 (377/414)	0.96
PPV	83 (74/89)	67 (74/111)	< 0.001^b^	78 (75/96)	76 (82/108)	0.37
NPV	96 (305/318)	96 (271/283)	0.89	98 (300/306)	96 (295/306)	0.06
Infra-popliteal arteries						
Sensitivity	95 (137/144)	99 (138/140)	0.04^a^	94 (142/151)	95 (143/150)	0.33
Specificity	92 (186/203)	92 (176/192)	0.99	99 (206/208)	99 (199/202)	0.53
Accuracy	93 (323/347)	95 (314/332)	0.44	97 (348/359)	97 (342/352)	0.76
PPV	89 (137/154)	90 (138/154)	0.86	99 (142/144)	98 (143/146)	0.57
NPV	96 (177/184)	99 (175/177)	0.04^a^	96 (206/215)	97 (199/206)	0.38
Overall						
Sensitivity	91 (220/242)	94 (221/236)	0.08	89 (227/254)	93 (237/255)	0.03^a^
Specificity	94 (566/602)	89 (514/576)	< 0.01^b^	96 (585/609)	95 (571/604)	0.01^b^
Accuracy	93 (786/844)	91 (735/812)	0.02^b^	94 (812/863)	94 (808/859)	0.96
PPV	86 (220/256)	78 (221/283)	< 0.01^b^	90 (227/251)	88 (237/270)	0.07
NPV	96 (539/560)	98 (511/524)	0.17	96 (585/612)	97 (571/589)	< 0.01^a^

Data are given as percentages; numerators and denominators are displayed in parentheses

*PPV* positive predictive value, *NPV* negative predictive value

^a^Indicates a statistically significant advantage of axial images over mpCPRs; ^b^indicates a statistically significant advantage of mpCPRs over axial images; ^c^could not be calculated

Different stages of PAD had no significant effect on the diagnostic accuracy of the less-experienced reader (*p* = 0.86), and the difference in diagnostic accuracy between axial images and mpCPRs was not affected by the clinical stage of PAD (*p* = 0.16). The same applies for the expert reader (*p* = 0.99 and *p* = 0.65, respectively).

In particular, the less-experienced reader reached an overall accuracy of 92 and 91% with mpCPRs and axial images for patients with critical limb ischemia, respectively. For patients with claudication, he reached an accuracy of 94 and 90% with mpCPRs and axial images, respectively. The expert reader reached an overall accuracy of 94% for all patients, independent of clinical stage and imaging technique used.

Overall inter-reader agreement was excellent for both mpCPRs and axial images, with 92 and 90% agreement, respectively. The inter-reader agreement showed no significant difference between mpCPRs and axial images (*p* = 0.254). Detailed inter-reader agreement values for each vascular territory are provided in Table 3.

**Table 3:** Inter-reader agreement for CT angiography according to vascular region

	mpCPR	Axial	*p* value
Iliac arteries	94 (85/90)	88 (76/86)	0.096
Femoro-popliteal arteries	89 (359/405)	86 (335/389)	0.121
Infra-popliteal arteries	93 (324/344)	94 (312/330)	0.869
Overall	92 (768/839)	90 (725/805)	0.254

Data are given as percentages; numerators and denominators are displayed in parentheses

Of note: due to different segments excluded by both readers, the denominator values differ from those in Table 2

### Time Efficiency

Both readers were significantly faster when using mpCPRs than when using axial images (Reader 1: *p* < 0.01, Reader 2: *p* < 0.01). Reader 1 was, on average, 25% faster with mpCPRs (mean evaluation time 5:45 ± 1:42, range 1:42–9:25 min) than with axial images (mean evaluation time 7:40 ± 2:02, range 3:26–12:15 min). Reader 2 was, on average, 33% faster, when the evaluation was based on mpCPRs (4:41 ± 1:20, range 1:24–7:42 min) compared to axial images (6:57 ± 1:42, range 3:26–10:27 min).

## Discussion

The main finding of our study is the high diagnostic accuracy of mpCPRs for the assessment of PAD. Whereas the expert reader reached a similar level of accuracy using either axial images or mpCPRs, the diagnostic accuracy of the less-experienced reader was even higher with mpCPRs. This is in contrast to studies on other 3D reformation techniques, such as MIP and STVR, which failed to reach the diagnostic accuracy of axial images when used exclusively [18, 19]. With regard to specificity, MIPs have been shown to be clearly inferior to axial images as well [19]. In this study, however, the specificity of mpCPRs significantly surpassed that of axial images. Only the sensitivity of mpCPRs was, similar to STVRs [19], inferior to that of axial images. Overall inter-reader agreement for mpCPRs was slightly higher than that of axial images, confirming the robustness of this reformation technique.

**Fig. 3:**
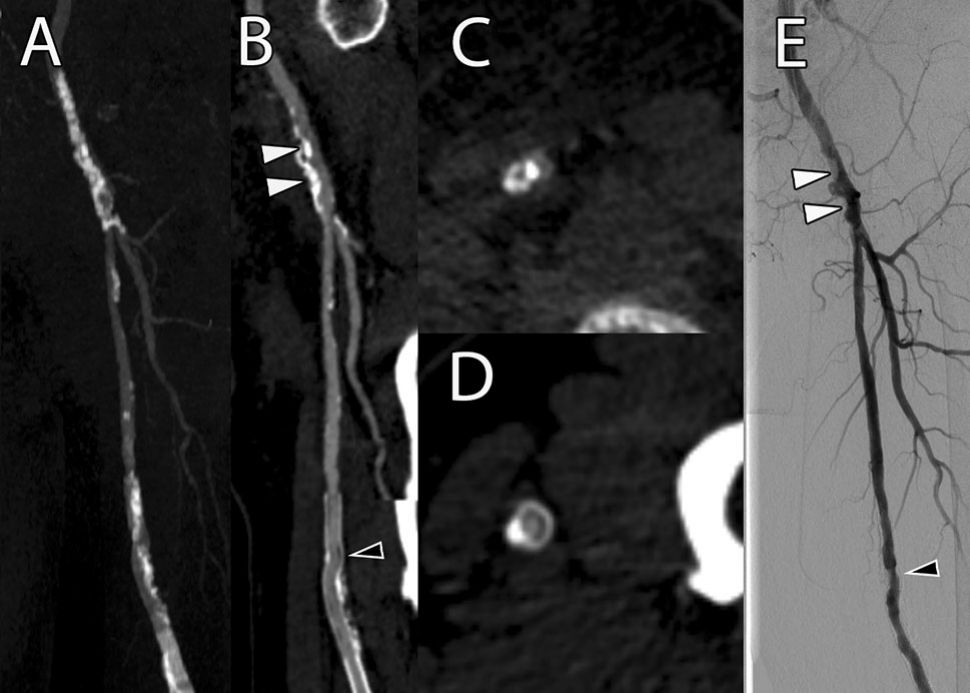
A 76-year-old male patient with diabetes type II and a history of stent implantation in the left superficial femoral artery was referred to interventional radiology with PAD category 3 according to the Rutherford classification. **A** MIP cannot assess the vessel lumen in the presence of severe vessel wall calcifications or stents. **B** mpCPR clearly showed severe calcifications in the left common femoral artery, causing a significant stenosis (proximal white arrowhead), followed by a moderate tandem stenosis (distal white arrowhead), as well as moderate neo-intimal hyperplasia in the stent of the left superficial artery (black arrowhead). **C** Axial image of the left common femoral artery at the level of the proximal white arrowhead in **B** showed a significant stenosis as well. **D** Axial image of the left superficial femoral artery at the level of the black arrowhead in **B** showed a moderate in-stent-stenosis as well. **E** DSA confirmed the findings of mpCPRs and axial images

As previously reported, mpCPRs provide a significantly greater viewing range of the peripheral arterial tree than MIPs [22]. This might be one explanation for the superior diagnostic performance of mpCPRs in this study, compared to the previously reported performance of MIPs [19]. The second advantage of mpCPRs over MIPs is their capability to visualize the vessel lumen inside stents or in the presence of severe vessel wall calcifications, as demonstrated in Fig. 3. Interestingly, both readers declared more vessel segments too calcified for evaluation on axial images than on mpCPRs. This was especially true for vessels below the knee, where the vessel diameter is represented by a few voxels only, so that the identification of contrast media next to severe calcifications can be difficult on axial slices, whereas reformatted images facilitate the assessment of the contrast media continuity along the vessel path.

**Fig. 4:**
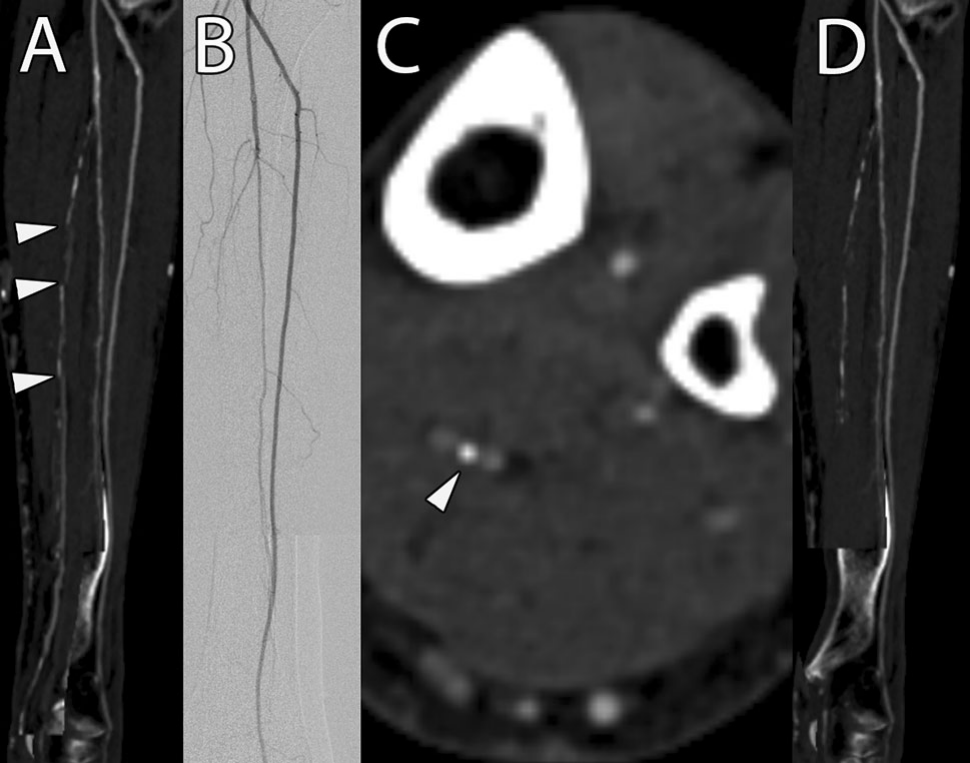
An 85-year-old female patient was referred to interventional radiology due to PAD stage category 2 according to the Rutherford classification. During the evaluation of mpCPR images, Reader 1 reported the left posterior tibial artery as patent (**A**), although it was occluded on DSA (**B**). Reader 2 recognized that the automatic vessel tracking of the software misinterpreted the venous overlay of the accompanying veins as the correct vessel path due to the occlusion of the calcified posterior tibial artery (white arrowhead in **C**). Unfortunately, the technician in charge did not realize this faulty automated tracking during the generation of the reformations, although it could have been identified by the step formations caused by the switching between artery and accompanying vein (white arrowheads in **A**). **D** shows a corrected reformation, which was generated by the expert radiologist after the analysis of the study results

Interestingly, below-the-knee arteries, in particular their limited number of orthogonal viewing pairs observed in an initial analysis of mpCPRs in 2007, were the reason for the recommendation to use mpCPRs not alone, but rather in conjunction with spCPRs, MIPs, and axial images [22]. Looking at the results of the less-experienced reader only, this recommendation might seem to be confirmed, as he reached a significantly lower sensitivity with mpCPRs for the infra-popliteal arteries, but not for the iliac or femoro-popliteal arteries. However, taking a closer look at Table 2, the less-experienced reader excluded more vessel segments on axial images than on mpCPRs due to low contrast or prosthesis-related artifacts, whereas the absolute number of stenosed segments was almost the same on mpCPRs (*n* = 137) and on axial images (*n* = 138). The expert reader, on the other hand, showed no significant difference between mpCPRs and axial images in any statistical test, for the infra-popliteal arteries. Therefore, we hypothesized that the higher number of false-negative findings below the knee for the less-experienced reader might be attributed to some cases with occluded infra-popliteal arteries and early enhancement of the accompanying veins. In these cases, the automatic vessel tracking algorithm can misinterpret the venous overlay as the correct vessel path. Figure 4 shows an example in which the more-experienced reader correctly identified the faulty reformation and rated the vessel as occluded, whereas the less-experienced reader rated the vessel as patent. This faulty reformation should have been identified and corrected during the image post-processing, which clearly shows that this task must be diligently performed to leverage the full strength of this reformation technique.

Although we showed that mpCPRs of the infra-popliteal arteries can comprise pitfalls for less-experienced readers, this reformation technique was advantageous, especially for the less-experienced reader. He reached a higher level of specificity and accuracy than that reached with axial images, overall, and, in particular, in the femoro-popliteal arteries, while maintaining a similar level of sensitivity.

Furthermore, the reading of mpCPRs was statistically significantly faster compared to that of axial images, although the absolute time saving was smaller than anticipated. In particular, for the less-experienced reader, a more pronounced effect was expected. An explanation for the smaller difference might be the sub-segments that were defined for long vessels in this study. For each sub-segment, a separate rating was necessary and the allocation of stenoses to the correct segment required additional time for both axial images and mpCPRs. Of note, technologists routinely perform the image post-processing at our institution and the mpCPRs are stored in the PACS. In other institutions, where radiologists are performing image post-processing themselves, the required post-processing time would exceed the time savings of mpCPRs during image analysis. However, in addition to being faster with the mpCPR images, both readers reported that the reading of axial images was more tiring. In theory, this could lead to an increased number of careless mistakes during the stressful clinical routine [13]. Besides time savings during the diagnostic workup of PAD, the angiogram-like overview of the peripheral vascular tree provided by mpCPRs facilitates the assessment of all treatment-relevant parameters [14, 23]. Thus, they have become the preferred imaging modality for treatment planning in our interdisciplinary vascular board.

There are certain limitations to this study that need to be addressed. First, the DSA procedure was not specifically modified for study reasons due to ethical considerations about radiation exposure. Therefore, DSA was not obtained for all segments. However, this was considered in the initial power analysis. In addition, most vessel segments were visualized by a single plane of DSA; a second plane was only acquired if clinically indicated during the procedure. Second, none of our patients had a femoro-femoral crossover bypass, which causes severe artifacts in mpCPRs due to their path running parallel to the horizontal axis [22]. We recommend CTA for the follow-up of such bypasses, to be assessed on axial images or coronal reformations. Third, the prototype software used requires dedicated hardware and needs to be recompiled for each workstation; thus, it is not commercially available. However, a web-based application with a server processing the datasets in the background is currently being developed and will be made available once completed.

In conclusion, mpCPRs are a promising reformation technique that facilitates a fast assessment of PAD with high diagnostic accuracy. However, radiologists need to be aware of possible reformation-related pitfalls in case of occluded infra-popliteal arteries with venous overlay.

## Electronic Supplementary Material

A 75-year-old male patient with hypertension and hyperlipidemia and a history of stent implantation in both superficial femoral arteries was referred to interventional radiology with PAD category 2 according to the Rutherford classification. 21 Maximum Intensity Projections (supplemental video 1) and 21 Multipath Curved Planar Reformations (supplemental video 2) were rendered over a viewing range of 180° in 9° intervals.

Supplementary material 1 (MP4 2205 kb)

Supplementary material 2 (MP4 1971 kb)
